# Drug education in victorian schools (DEVS): the study protocol for a harm reduction focused school drug education trial

**DOI:** 10.1186/1471-2458-12-112

**Published:** 2012-02-10

**Authors:** Richard Midford, Helen Cahill, David Foxcroft, Leanne Lester, Lynne Venning, Robyn Ramsden, Michelle Pose

**Affiliations:** 1 Edith Cowan University, Perth, Australia; 2 The University of Melbourne, Melbourne, Australia; 3 Victorian Department of Education and Early Childhood Development, Melbourne, Australia; 4 Oxford Brookes University, Oxford, UK

**Keywords:** Prevention, Drug education, Harm reduction, Schools, Students

## Abstract

**Background:**

This study seeks to extend earlier Australian school drug education research by developing and measuring the effectiveness of a comprehensive, evidence-based, harm reduction focused school drug education program for junior secondary students aged 13 to 15 years. The intervention draws on the recent literature as to the common elements in effective school curriculum. It seeks to incorporate the social influence of parents through home activities. It also emphasises the use of appropriate pedagogy in the delivery of classroom lessons.

**Methods/Design:**

A cluster randomised school drug education trial will be conducted with 1746 junior high school students in 21 Victorian secondary schools over a period of three years. Both the schools and students have actively consented to participate in the study. The education program comprises ten lessons in year eight (13-14 year olds) and eight in year nine (14-15 year olds) that address issues around the use of alcohol, tobacco, cannabis and other illicit drugs. Control students will receive the drug education normally provided in their schools. Students will be tested at baseline, at the end of each intervention year and also at the end of year ten. A self completion questionnaire will be used to collect information on knowledge, patterns and context of use, attitudes and harms experienced in relation to alcohol, tobacco, cannabis and other illicit drug use. Multi-level modelling will be the method of analysis because it can best accommodate hierarchically structured data. All analyses will be conducted on an Intent-to-Treat basis. In addition, focus groups will be conducted with teachers and students in five of the 14 intervention schools, subsequent to delivery of the year eight and nine programs. This will provide qualitative data about the effectiveness of the lessons and the relevance of the materials.

**Discussion:**

The benefits of this drug education study derive both from the knowledge gained by trialling an optimum combination of innovative, harm reduction approaches with a large, student sample, and the resultant product. The research will provide better understanding of what benefits can be achieved by harm reduction education. It will also produce an intervention, dealing with both licit and illicit drug use that has been thoroughly evaluated in terms of its efficacy, and informed by teacher and student feedback. This makes available to schools a comprehensive drug education package with prevention characteristics and useability that are well understood.

**Trial registration:**

Australia and New Zealand Clinical Trials Register (ANZCTR): ACTRN12612000079842

## Background

The health and social costs associated with alcohol and other drug (AOD) use are considerable and fall most heavily on young people [1-3]. This in itself is an argument for early AOD prevention programs. School drug education offers the potential to prevent problems by equipping young people with the knowledge and skills to make responsible decisions about AOD use, and it has near universal reach in developed countries where the great majority of young people attend secondary school [4]. However, historical approaches to school drug education have not been particularly successful at reducing AOD use [5-7]. This then poses the question as to whether effectiveness should be measured by abstinence or reduced use, or whether harm reduction is a more realistic and useful measure. Harm reduction programs offer greater promise of achieving worthwhile benefit because they have the flexibility to select strategies on the basis of evidence of effect. Within this model abstinence or reduced use strategies may be chosen if there is evidence that they reduce harm, but they are not goals in their own right [8].

The School Health and Alcohol Harm Reduction Project (SHAHRP) demonstrated the effectiveness of a skills based, harm reduction intervention. Students who received the SHAHRP program experienced 22.9% less alcohol-related harm than their control peers [9]. A more recent study of computerised harm reduction prevention similarly reported that alcohol consumption, risky drinking and alcohol-related harms increased to a lesser extent among female intervention students, although there was no program effect among male students [10]. A related study that targeted cannabis, as well as alcohol, found that weekly alcohol consumption of intervention students decreased, while that of their control peers increased. The frequency of drinking to excess also increased to a lesser extent among the intervention students [11].

Drug education has developed considerably in Australia over the past decade. However, evidence-based practice tends to be best reflected in demonstration programs that focus on a single drug type, such as alcohol [9,12]. The most recent findings have not been brought together in a mass drug education program that targets all forms of drug use and can be readily accommodated within a secondary school health curriculum. Past classroom-based programs have not well exploited the important influence that parents have on their children in terms of AOD choices and the likely benefits of increased communication on this issue [13]. Finally, while most Australian school drug education programs are grounded in the research literature and principles of effective practice there has been insufficient attention to the pedagogy required to deliver effective classroom lessons [14]. Drug education is most effective when inclusive, interactive teaching strategies actively engage students in the learning process [14,15]. However, reviews of drug education program implementation consistently identify a breakdown in fidelity when learning tasks are interactive [16-18].

This research study seeks to extend earlier Australian harm reduction education research by investigating the prevention effects of a single program for both licit and illicit drugs. The size and nature of the intervention have been tailored to facilitate incorporation within an already crowded school curriculum. It draws on the recent literature as to the common elements in effective school drug education programs [19-22]. It seeks to incorporate the social influence of parents through structured home activities that complement the classroom lessons. It emphasises the use of appropriate pedagogy through two days of training, where participatory delivery of each lesson is modelled to ensure teachers are equipped to teach the program as intended. All these elements have been trialled and refined during the course of a two year pilot program that was undertaken in four Victorian government secondary schools (three intervention and one control) [23].

### Conceptual underpinnings

The intervention at the heart of this study is grounded in social learning theory, which posits that human learning occurs in a social context [24]. In this model, drug use is socially learned through modelling, imitation and reinforcement, and influenced by an individual's cognitions, attitudes, and beliefs. The corollary is that drug education can use the same learning processes to equip students with the skills to recognise these influences and develop a repertoire of counter behaviours. Social learning theory underpins all of the most effective school drug education programs, and a number of researchers have indicated that it should be the model of choice in any prevention program [7,19,25].

The intervention also draws on two other theoretical models, poststructuralist subjectivity and cognitive dissonance. Poststructuralist subjectivity theory provides a way of understanding how students' self concepts, and hence their approach to drug use, can change. Cognitive dissonance theory provides a way of understanding how students resolve conflicting ideas on drug use.

Poststructuralist subjectivity theory refers to the ways in which the social world is involved in shaping an individuals' sense of self [26,27]. It posits that cultural norms and expectations shape the individual's sense of who they can or should be, and identifies the importance of a 'sense of belonging' to identity formation [28]. In this process of subjectification, identity is continuously under construction, maintenance and re-construction, and hence open to change, particularly change that is social and collective in nature. Thus participatory and collective learning methods become the vehicle for the creation of new norms and possibilities in the peer group [14,15,29].

Festinger's theory of cognitive dissonance posits that contradictory cognitions, or cognitive dissonance, cause mental discomfort [30]. This serves as a driving force that compels a person to adopt new thoughts or beliefs, or to modify existing beliefs, so as to reduce the amount of dissonance between cognitions. The education intervention used in this study does not tell students what they should think or how they should behave in terms of drug use. Rather, it provides a social learning process whereby the students reach their own conclusions through exploration of drug use issues. By guiding students through an interactive discovery process that involves articulation of responsible drug use behaviour their ownership of that behaviour is reinforced and they are less likely to behave in a contrary manner.

### Aims and hypothesis

The aim of this study is to develop and assess the effectiveness of a comprehensive, evidence-based, harm reduction focused school drug education program for year eight and nine students by undertaking a cluster randomised trial with 21 secondary schools in the Victorian state of Australia. The specific hypothesis is that while the intervention students may not be any less likely to take up use of alcohol, tobacco and illicit drugs they will be more likely to consume in a less risky manner and experience less harms associated with use.

## Methods/design

### Study design

Twenty one Victorian government high schools have been recruited to the study. The schools were then allocated to four strata: six schools to metro location, high SES; three schools to rural location, high SES; six schools to metro location, low SES; six schools to rural location, low SES. This approximated the proportion of Victorian secondary schools in each category. Within each strata the schools were then randomly allocated to intervention or control conditions on a two to one proportion to allow more precise statements about the effects of the intervention [31]. Random allocation and allocation concealment were achieved by one member of the research team writing the names of each school on identical paper chits which were then folded to conceal the name. The same researcher also created seven chits with the word control and 14 with the word intervention. These were similarly folded to conceal the words. The chits with the school name and those designating allocation were placed in separate containers. A different member of the team then simultaneously drew out a chit with the name of a school and a chit designating either control or intervention allocation.

**Figure 1:**
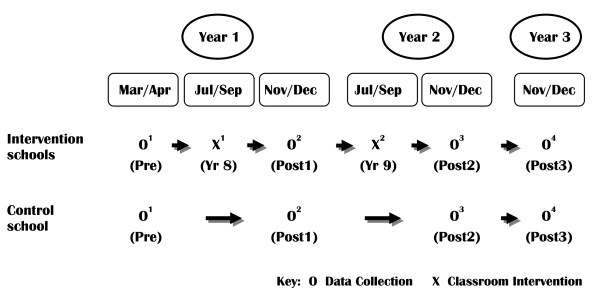
**Schematic illustration of the research design**.

The study will follow a cohort of students from the start of year eight, when most are 13 years old, to the end of year ten, when most would have turned 16. Pre intervention, baseline testing will occur at the beginning of year eight (Mar/Apr). The first phase of the drug education intervention will occur in the middle of that year (Jul/Sep). Post 1 testing will occur at the end of the same year (Nov/Dec), approximately 8 months subsequent to baseline. The second phase of the drug education intervention for the students, now in year nine, will occur in the middle of the second year of the study. Post 2 testing will occur at the end of the second year. In the third year, when the students are in year ten, no further drug education lessons will be provided as part of this study, but schools will continue with their usual year ten drug education curriculum. Post 3 testing will occur at the end of the year. Throughout the study control students will receive the drug education normally provided by their school. The research sequence is illustrated in Figure 1. Fidelity data will be collected from all participating teachers on a lesson by lesson basis via an on-line survey. In addition, focus groups/interviews will be used to collect qualitative information about the relevance and suitability of lesson materials from students and teachers in a sample of five of the 14 intervention schools following delivery of both the year eight and the year nine program. The student focus groups will be one hour in duration with a roughly even mix of six to eight males and females. Teacher interviews will also be one hour long with either individuals, or small groups of two to three respondents.

### Sample size calculation

The ability to detect small changes in substance use, knowledge, attitudes and harms experienced is central to this study. The primary outcome is a change in alcohol consumption patterns and associated harm, and accordingly sample size estimations are based on detecting a small effect size of .15 in relation to these measures. This effect size was chosen on the basis of previous school drug education studies [9,32].

The target sample size has been estimated using G*Power v.3.1.3 software where α = 0.05 and 1-β error probability = 0.95 [33]. Assuming simple random sampling, a total sample size of 364 is required at the end of the study. However, there is a design effect due to the loss of effectiveness created by cluster sampling. The design effect for the SHAHRP study, which took into consideration the effect of clustering by school and a 15% annual rate of student attrition, was calculated to be 1.48. Using this correction, a total sample size of at least 539 is required to test the effectiveness of the intervention.

### Sample

The participating students attended the 21 Victorian government secondary schools that had agreed to participate in this project. Four schools are located in metropolitan Melbourne; ten are located in metropolitan fringe and major regional areas; and seven are in regional and rural areas. The student populations of six schools are in the medium high/high range in term of socioeconomic status, as measured by the Department of Education and Early Childhood Development's (DEECD) Student Family Occupation (SFO) index. The student populations of ten schools are in the medium range. The student populations of five schools are in the low range. Written active consent was sought from the 2700 year eight students in the 21 participating schools and their parents. Of this total population 1746 or 64.7% agreed to participate in the research. Intervention students numbered 1230. Control students numbered 516.

### The drug education intervention

The student intervention material and an accompanying teacher implementation manual and teacher training program have been developed from material trialled in the pilot program [23], which in turn drew on a range of earlier Australian research and development projects in drug education and resilience education. These included the School Health and Alcohol Harm Reduction Project (SHAHRP) and GET WISE: Working on Illicits in School Drug Education [9,34]. The education program comprises 10 lesson plans in year eight and 8 in year nine that address issues around the use of alcohol, tobacco, cannabis and other illicit drugs (see Table 1). Experience from the SHAHRP study indicated that this number of lessons can provide adequate coverage and be accommodated within a secondary school curriculum. A number of tasks are designed to be undertaken at home in collaboration with a parent so as to draw out their influence on decision-making about drug use [35]. As alcohol is the most commonly used drug by Australian young people, and the drug that causes them the greatest harm, it will receive the greatest coverage, followed by tobacco and cannabis.

**Table 1:** Year eight and year nine lesson plans

Lesson	Year 8	Year 9
1	WHAT IS A DRUG? - Introduction, agreements, definitions and drug categories	PRIORITIES AND CONCERNS - Identifying what young people value and worry about and what worries they have around drugs

2	ALCOHOL AND EFFECTS AND STANDARD DRINKS - How alcohol effects the body, assessing harms associated with use, pouring standard drinks, understanding blood alcohol content and safer levels of use	FACING FACTS AND FINDING SOLUTIONS - Alcohol and Cannabis- guidelines on use and the research that informs them

3	PARTY BEHAVIOURS AND ALCOHOL - The relationship between levels of alcohol use and the risk of harm to self and others	USING YOUR RESOURCES - Pouring standard drinks, matching harms to levels of alcohol use, identifying strategies to reduce harm

4	PREVALENCE AND NORMS - Dispelling myths about levels of drug use amongst young people, identifying reasons for use/non -use	WINDING UP, WINDING DOWN - Learning about the effects/risks of Amphetamine type stimulants, identifying drug-free ways of achieving 'high' and 'serene' states of mind

5	TOBACCO - Considering gender differences in relation to smoking; the impact of media messages	DRUGS, DISINHIBITION, SEXUAL VULNERABILITY AND VIOLENCE - Discussing sexual vulnerability in relation to drug use, identifying strategies for avoiding or reducing harm

6	CANNABIS - Information about cannabis and its effects, identifying risks associated with Cannabis use	INVISIBLE RISKS - Information about injecting drug use, blood-borne viruses and methods of protection

7	RISK REDUCTION - Assessing risk and developing strategies to avoid or minimise harm	PERSONAL CONFIDENCE - AND DRUG USE - Developing and rehearsing positive self talk, refusal skills and tactics for peer negotiation

8	INFLUENCES - Identifying social and media influences to use alcohol	GETTING HELP AND TALKING WITH ADULTS - Information about heroin, rehearsing steps for practical first aid in situations involving overdose, rehearsing help seeking with adults

9	OPTIONS AND DECISIONS - Generating and rehearsing strategies to reduce harms associated with drug use	

10	STANDING UP FOR YOURSELF - Providing peer support, using assertion skills in situations involving alcohol	

The education program incorporates the elements of effective practice identified by reviews of school drug education [19-22,25,36]. This literature identifies the importance of basing programs on evidence of proven effect; the needs of students; provision of essential knowledge; adequate coverage of salient issues; and use of interactive learning strategies that enhance negotiation skills, involve participants in problem-solving and engage them in deconstructing the social pressures and perceived norms around drug use. The curriculum is also informed by research in the field of resilience education that identifies social competence, problem-solving, autonomy and a sense of purpose as key attributes of resilient young people [37], and highlights the importance of participatory and developmentally appropriate learning strategies in enhancing social and emotional learning [38].

Each year all teachers delivering the classroom program will participate in an intensive two-day professional learning program that provides a grounding in the evidence-base informing the study and active sampling of each of the lesson activities they will be teaching their students. Emphasis will be given to modelling and explicit leadership coaching in use of the participatory methods.

### Student survey instrument and measurement of change

The survey instrument to measure change is a development of the self-completion questionnaire used in SHAHRP and was trialled in the pilot research that preceded this study [9,23]. Self-report is well accepted practice in studies of this type and research indicates little inconsistency between self report and other measures of drug use [39,40] The instrument will collect information on knowledge, patterns and context of use, attitudes and harms experienced in relation to alcohol, tobacco, cannabis and other illicit drug use. As was done in the pilot study, scales will be constructed to measure overall change in AOD knowledge (38 items), attitudes (4/5 items per drug type), and harms (5/10 items per drug type)

A student generated code, based on easily remembered fragments of personal information, is used to maintain confidentiality, while allowing individual matching over the course of the study. As part of the pilot research, feedback was obtained from experts in school drug education and students in the target group. This indicated the instrument had both content and face validity. The Cronbach's alpha test was used to measure the internal consistency of the AOD knowledge, attitude and harm scales generated from the pilot survey responses. The results for the pilot AOD knowledge and alcohol, tobacco, cannabis and other drugs attitude and harm scales are presented in Table 2. The internal consistency co-efficient of all indices was significant. In the case of the knowledge scale and all the harm scales the respective co-efficients were high (α = .609-.949), indicating each scale was a good measure of the intended single latent construct [41]. The attitude scale co-efficients were lower, although still significant. Factor analysis of the attitudes towards alcohol, tobacco and other drugs indicated there were two main factors in the data accounting for between 53.5% and 57.9% of the total variation in each case. One factor pertained to attitudes about knowledge and communication; the other pertained to attitudes about harm.

**Table 2:** Internal consistency of knowledge, attitude and harm scales

Scale	alpha	p	Component factors	% of variance
Knowledge scale	.859	< 0.001		

Attitudes towards alcohol	.387	< 0.001	Knowledge and communication	30.6

			Harm	23.5

Alcohol harm scale	.949	< 0.001		

Attitudes towards tobacco	.445	< 0.001	Harm	32.4

			Knowledge and communication	21.1

Tobacco harm scale	.802	< 0.001		

Attitudes towards cannabis	.548	< 0.001		

Cannabis harm scale	.891	< 0.001		

Attitudes towards other drugs	.284	< 0.001	Knowledge and communication	32.7

			Harm	25.2

Other drugs harm scale	.609	< 0.001		

### Blinding

Participants cannot be blinded to intervention in this sort of psycho-social educational intervention, nor can program deliverers or outcome assessors.

### Research ethics

The study was approved by Edith Cowan University's and the University of Melbourne's human research ethics committees. It was also approved by the Research Branch, Education Policy and Research Division of the Victorian Department of Education and Early Childhood Development.

### Statistical analysis

Multi-level modelling can best accommodate hierarchically structured data and will be used in this analysis, adjusting for any baseline differences between the intervention and control groups [42,43]. The hierarchy in this longitudinal multi-level dataset comprises level 1 units (occasions of repeated measurement), nested within the level 2 units (the individual student), nested within the level 3 units (the school). Stata version 10.0 will be used for model fitting, with a three-level mixed regression model fitted to the data to account for the repeated observations [44]. If necessary, the knowledge, consumption and harm scales will be log-transformed to satisfy the assumption of normality. If normality cannot be achieved by log-transformation, non-parametric procedures will be used for analysis. All analyses will be conducted on an Intent-to-Treat basis. Complete-case analysis (CCA) will be complemented with multiple imputation analysis (MIA) to account for missing data.

## Discussion

The benefits of undertaking this harm reduction focused drug education demonstration program derive both from the knowledge gained by trialling an optimum combination of evidence derived approaches with a large, representative student sample, and the resultant product. The program targets alcohol, tobacco and illicit drug use; draws on research that consistently identifies a core set of effective practice elements and is rigorously evaluated over an extended period. The program also does not simply rely on the traditional measures of effectiveness, namely abstinence or reduced use. Harms associated with use are also measured as valid indicators of program effect. This will provide a broader understanding of what benefits can be achieved by school drug education. By the end of the study a well documented, comprehensive drug education package will have been produced. It will be of a size able to be accommodated within a secondary school's health curriculum and the evaluation findings will provide evidence as to its potency. This makes available to schools a readily useable drug education program with well understood prevention characteristics.

## Competing interests

The authors declare that they have no competing interests.

## Authors' contributions

RM obtained funding for the study, co-ordinated the design of the study, contributed to the design of the intervention materials and was the lead author of this study protocol. HC co-ordinated the design of the intervention materials and contributed to the study design and the drafting of this study protocol. DF contributed to the study design and the drafting of this study protocol. LL contributed to the study design and the drafting of this study protocol. LV recruited the study schools and contributed to the study design, the design of the intervention materials and the drafting of this study protocol. RR contributed to the study design, the design of the intervention materials and the drafting of this study protocol. MP contributed to the design of the intervention materials and the drafting of this study protocol. All authors read and approved the final manuscript

## Pre-publication history

The pre-publication history for this paper can be accessed here:

http://www.biomedcentral.com/1471-2458/12/112/prepub
